# Cities and neuroscience research: A systematic literature review

**DOI:** 10.3389/fpsyt.2022.983352

**Published:** 2022-11-10

**Authors:** Leonardo A. Ancora, Diego Andrés Blanco-Mora, Inês Alves, Ana Bonifácio, Paulo Morgado, Bruno Miranda

**Affiliations:** ^1^Institute of Physiology, Lisbon School of Medicine, University of Lisbon, Lisbon, Portugal; ^2^Institute of Molecular Medicine, Lisbon School of Medicine, University of Lisbon, Lisbon, Portugal; ^3^Centre of Geographical Studies, Institute of Geography and Spatial Planning, University of Lisbon, Lisbon, Portugal

**Keywords:** urban science, built environment, natural environment, neuroscience, brain research

## Abstract

**Background:**

Cities are becoming the socio-economic hubs for most of the world’s population. Understanding how our surroundings can mentally affect everyday life has become crucial to integrate environmental sustainability into urban development. The present review aims to explore the empirical studies investigating neural mechanisms underlying cognitive and emotional processes elicited by the exposure to different urban built and natural spaces. It also tries to identify new research questions and to leverage *neurourbanism* as a framework to achieve healthier and sustainable cities.

**Methods:**

By following the PRISMA framework, we conducted a structured search on PubMed, ProQuest, Web of Science, and Scopus databases. Only articles related to how urban environment–built or natural–affects brain activity through objective measurement (with either imaging or electrophysiological techniques) were considered. Further inclusion criteria were studies on human adult populations, peer-reviewed, and in English language.

**Results:**

Sixty-two articles met the inclusion criteria. They were qualitatively assessed and analyzed to determine the main findings and emerging concepts. Overall, the results suggest that urban built exposure (when compared to natural spaces) elicit activations in brain regions or networks strongly related to perceptual, attentional, and (spatial) cognitive demands. The city’s-built environment also triggers neural circuits linked to stress and negative affect. Convergence of these findings was observed across neuroscience techniques, and for both laboratory and real-life settings. Additionally, evidence also showed associations between neural social stress processing with urban upbringing or current city living–suggesting a mechanistic link to certain mood and anxiety disorders. Finally, environmental diversity was found to be critical for positive affect and individual well-being.

**Conclusion:**

Contemporary human-environment interactions and planetary challenges imply greater understanding of the neurological underpinnings on how the urban space affects cognition and emotion. This review provides scientific evidence that could be applied for policy making on improved urban mental health. Several studies showed that high-quality green or blue spaces, and bio-diverse urban areas, are important allies for positive neural, cognitive, and emotional processes. Nonetheless, the spatial perception in social contexts (e.g., city overcrowding) deserves further attention by urban planners and scientists. The implications of these observations for some theories in environmental psychology and research are discussed. Future work should take advantage of technological advancements to better characterize behavior, brain physiology, and environmental factors and apply them to the remaining complexity of contemporary cities.

## Introduction

Over the past few centuries, cities have become the cultural and political centers of most societies, and the scaffolding supporting changes in human desires, needs, and challenges (1, 2). In 2018, more than half of the world’s population lived in urban areas and it is estimated that by 2050 the number will rise to around two-thirds (3). Such urbanization phenomenon is, as endorsed by the United Nations General Assembly (4), one of the twenty-first century’s most transformative trends. The advantages of living in cities are spread across several socio-economic areas, from a better income and level of employment to a higher education and access to healthcare (5). However, city dwellers also face a vast array of inequalities or environmental changes (e.g., pollution of air and water, transportation problems, reduced social cohesion); and several urban features have direct and/or indirect bearing on human health, physical, and mental (6, 7).

Urban health is a developing discipline concerned with cities’ determinants of health and diseases, as well as with the city-living context as an exposure of interest (8)–being a good example of how challenging it could be to achieve sustainable urban development (9, 10). Nonetheless, rather than documenting unhealthy exposures and highlighting the complexity of the human-environment relationship, contemporary urban science examines these interactions at different levels and aims to fully address the causes and mechanisms. This is particularly relevant when studying the interdependence between city life and mental health and wellbeing, where a multi-disciplinary approach–involving biological, socio-economic, infrastructural, and cultural aspects–is crucial (11–15). Despite the vast epidemiological literature emphasizing the association between mental disorders and urban life (16), as well as the advances on how city living and urban upbringing affect the brain processing and associated stress (17), it remains elusive how urban life “gets under the skin” (14).

A person’s natural and built environments have a significant effect on the biological processing of cognitive and emotional information. People experience negative (fear, anger, disgust, and sadness) or positive (joy, trust, anticipation, and surprise) emotions while interacting with their surroundings. Research suggests that emotions are the driver of our most important decisions in life; similarly, our cognitive behavioral choices are the conduit for increasing positive emotions or decreasing negative emotions–tendencies associated with well-being (18). Moreover, the impact of cognition and emotions on health is experienced *via* physiological and behavioral mechanisms.

The brain, through complex neural circuits, is at the heart of our homeostatic control and the way we react to environmental stress. It determines what is threatening as well as the behavioral and physiological adaptive responses (19, 20). Neuroscience studies the human brain by focusing either on the effects of pathological changes (e.g., stroke, trauma, or other diseases), or by measuring cerebral activation during a particular behavioral task. The most widely used methodologies are non-invasive and aim to quantify neural activity across brain regions under well controlled conditions. Over the past few decades, we remarkably advanced our knowledge about the neural circuits and basic physiology of cognitive and emotional processing. Concomitantly, we have witnessed a wealth of new technologies, wearable devices, and software applications. Hence, neuroscience has not only moved from classic laboratory-based approaches to more real-world domains (21); but it has also extended its application into a variety of different fields–such as marketing (22), economics (23), and educational (24) sciences.

By recognizing similar potential benefits to the field of urban science, some authors have recently drawn attention to *neurourbanism*–an emerging discipline where theoretical perspectives and analytical methods of basic and clinical neuroscience support urban planning and design practice (11, 12, 25). Addressing the interdependencies between urbanization and neuroscience, it is possible to develop novel theoretical ideas and analytical methods critical for the creation of better city (built or natural) environments that will improve the mental (and physical) wellbeing of individuals and communities.

A noticeable progress has been made with respect to the link between architecture and the neural mechanisms of spatial navigation (26), aesthetics or design (27, 28). Some of this work aimed to address relevant theoretical formulations, including the cognitive and affective benefits of environmental enrichment (29) and the Appleton’s habitat theory (30)–for which an emotionally and aesthetically pleasing environment reflects its favorability to survival. Organizations (31) are promoting the knowledge that links neuroscience research to a growing understanding of human responses to the built environment.

Similarly, it is also paramount to support with neural evidence some prominent environmental psychology theories that aim to explain benefits of exposure to nature, such as the Attention Restoration Theory (ART) (32, 33)–suggesting that natural environments promote recovery from stress and fatigue *via* attention restoration mechanisms; and the Biophilia theory (35)–proposing that brain responses to natural settings reflect more pleasure and relaxation due to an evolutionary benefit of human affinity for nature.

In a systematic review performed a few years ago, Norwood and colleagues (34) explored how different environments affect brain activity and associated mood response. Their results indicate restorative feelings with natural environments and negative affect linked to urban environments. However, their analysis was restricted to mood and emotional processing–leaving cognitive domains aside. Further work (36, 37) also reviewed relevant human-built environment interaction, but their focus was limited to the architectural and interior design settings. In addition, a plethora of new studies have emerged since these reviews. Therefore, there is a clear research need to examine how cognitive neuroscience research has been applied to study the balance between natural ecosystems and built environments in urban areas. Moreover, a contemporary review should also fill a research gap by integrating the emerging evidence about the impact of urban upbringing and current city-living on brain processing.

**FIGURE 1:**
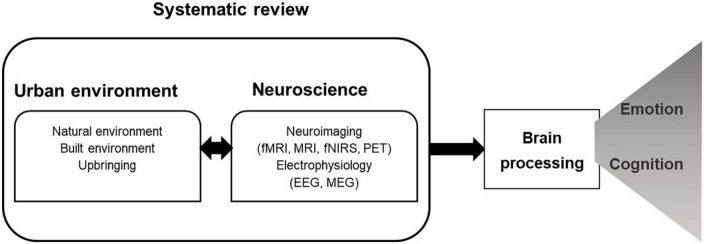
Conceptual model guiding the review.

Here, we aim to provide an up-to-date systematic review of empirical studies investigating the impact of urban built and natural exposure on brain activity and associated cognitive and emotional processes (see Figure 1). We explore the wide range of objective brain measurements collected throughout different contexts (in the laboratory or with outdoor real-life experiments), to highlight current explanatory strengths and identify future research needs. With this exercise, we specifically aim to:

1. Provide an up-to-date synthesis of the empirical evidence on how urban exposure and their modulators affect individual brain physiology.
2. Describe the methodologies and contexts that were most used for obtaining such knowledge.
3. Identify emerging concepts and key evidence-based knowledge both scientifically rigorous and capable of informing future research and policy-making agendas.

## Methods

The review is reported and follows the Preferred Reporting Items for Systematic Reviews and Meta Analyses (PRISMA) guidelines (38).

### Eligibility criteria

We considered all empirical studies written in English published in full-length peer-reviewed journals (abstracts, conference proceedings, and opinion/review articles were excluded but used to search for additional references) until 26th June 2021.

A “Population, Exposure, Comparator, and Outcomes (PECO) approach” (39) was used to define the eligibility criteria. We included studies where brain research methods were applied to human adult populations (healthy or diseased). To be included, studies had to specifically focus on examining how the exposure to urban built or natural environments influenced objective measures of brain activity (exposure duration was not restricted; representations of the environment using virtual reality, pictures or videos were accepted). Comparisons were not specified as criteria, and hence studies (cross-sectional or longitudinal; retrospective or prospective) where participants were exposed to more than two environments or exposed within a context of a randomized or non-randomized trial (e.g., crossover, parallel group, factorial) were considered. Included studies had to provide at least one quantitative outcome measure for brain activity (recorded either pre-, peri-, or post-exposure), either obtained through functional magnetic resonance (fMRI), functional near-infrared spectroscopy (fNIRS), positron emission tomography (PET), electroencephalography (EEG) or magneto-encephalography (MEG). Structural MRI was not included because our focus was on brain activation. Similarly, studies with only psychophysiological measurements (such as heart rate, skin conductance, pupil detection, or eye tracking) do not directly tap into the brain response and, therefore, were also not considered.

### Search strategy and information sources

The search strategy involved agreement between our inter-professional team coming from the neuroscience (basic and clinical) as well as urban planning and design fields. Four electronic databases were searched: PubMed, ProQuest, Web of Science, and Scopus. Search terms were related to both urban built and natural environments, as well as to quantitative research measuring brain activity effects of such exposures (see Table 1).

**TABLE 1:** Search terms used for the query.

Urban										Measurements
Strictly urban	OR	Natural			OR	Built			AND	Brain activity
Title/Abstract		Title/Abstract	AND	Title/Abstract		Title/Abstract	AND	Title/Abstract		Title/Abstract
urban outdoor urban space* urban environment* urban landscape* urban design urban planning urbanism urbanization urbanization urban form urban morphology urban infrastructure urban typology urban typologies urban renewal city city space city environment city landscape city design city planning city form cities public space urban public space urban centre urban inner circle historical centre city centre interior city urban expansion urban dynamic urban footprint urban life urban artifacts Urban nature		park fest tree garden		urban* city cities		Street neighbourhood neighbourhood building		urban* city cities		EEG Event related potential ERP magnetic resonance imaging MR MRI fMRI neuroimaging time resolved spectroscopy Near-infrared spectroscopy NIR NIRS Positron Emission Tomography PET MEG
		natural environment* natural infrastructure* natural space* natural landscape* natural outdoor* nature-based nature exposure nature contacts nature sound natural environment* natural setting* green space* greenspace* greenery blue space* bluespace*				built environment physical environment				

### Study selection and data collection process

**FIGURE 2:**
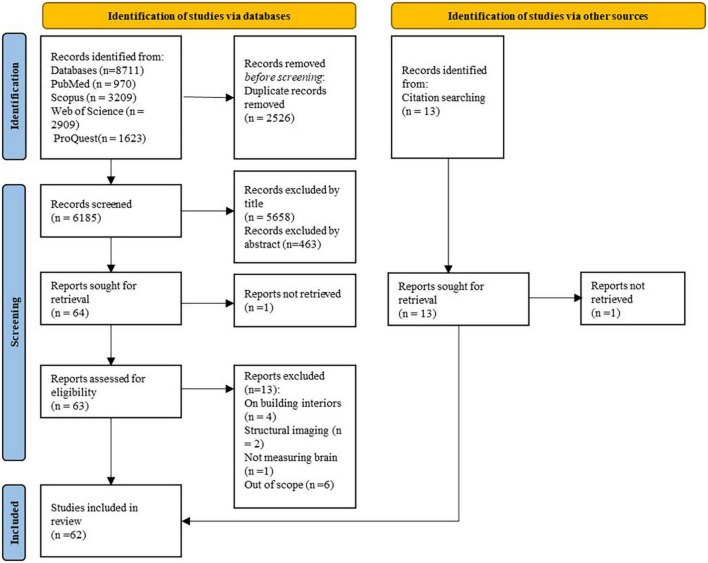
Preferred reporting items for systematic reviews and meta analyses (PRISMA) flow diagram of the selection process.

Initially, two different teams, with a neuro-scientific/medical (LAA, IA, BM) and urban/architectural (PM, AB) background, screened each paper independently according to titles and abstracts to identify an eligible subset. Then, an independent evaluation of the retained articles was firstly performed by two authors (LAA, IA) and then validated by the last author to ascertain eligibility and scan reference lists. Selected studies were then distributed among three authors (LAA, IA, BM) for a full-text review (see Figure 2), who independently extracted data into a pre-designed data extraction table (including information about the authors, publication date, objectives, study design and setting, population characteristics, sample size, environmental exposures, brain measurements, results summary, and major limitations). Discrepancies were resolved by team discussion. To ensure the screening process was accurate, 5% (169) of 3,380 titles and abstracts were randomly selected and cross validated among the teams to assess data reliability (the internal validation was good with a Kappa score of 78%).

### Quality appraisal and synthesis of evidence

We assessed the quality of included studies using a standard quality assessment criterion (40). Two authors independently scored (zero for no, one for partial, two for yes) on fourteen criteria (which address biases in sample selection, quantification of barriers, measure of the outcome, appropriateness of statistical analysis, or adjusting for confounders when applicable). Any disagreement was resolved by discussion. The sum of all scores was then divided by the highest possible score (see Table 2).

**TABLE 2:** Data extraction and quality assessment.

References	Author	Type of participant	Sample size	Age mean, SD	Measure	Environmental features	Setting	Quality score
[41]	Kim GW et al. 2010	Volunteer	28	26.9, 1.2	fMRI	Built and Natural environment	Indoor	0.91
[42]	Kim TH et al. 2010	Volunteer	30	27,3.7	fMRI	Built and Natural environment	Indoor	0.91
[43]	Seiyama et al. 2018	Volunteer	11	23.4,1.2	fMRI	Built and Natural environment	Indoor	0.86
[44]	Martínez-Soto 2013	Volunteer	28	36.18,12.46	fMRI	Built and Natural environment	Indoor	0.73
[46]	Kuhn et al. 2021	Volunteer	24	28.5, 9.5	fMRI	Built and Natural environment	Indoor	0.95
[47]	Bratman et al. 2015	Volunteer	38	26.7	fMRI	Built and Natural environment	Indoor	0.91
[48]	Tang et al. 2017	Volunteer	31	25	fMRI	Built and Natural environment	Indoor	0.86
[49]	Zhang et al. 2019	Volunteer	16	20.5,1.71	fMRI	Natural environment	Indoor	0.86
[50]	Chang et al. 2021	Volunteer	44	23.7	fMRI	Built and Natural environment	Indoor	0.91
[51]	Tost et al 2019	Volunteer	33	23.64,2.42	fMRI	Natural environment	Hybrid	0.86
[52]	Heller 2020	Volunteer	122	23,3.4	fMRI	Built and Natural environment	Hybrid	0.91
[17]	Lederbogen et al. 2011	Volunteer	55	N/A	fMRI	N/A	Indoor	0.77
[56]	Reed et al. 2020	Volunteer	487	31.9,8.9	fMRI	N/A	Indoor	0.82
[57]	Lemmers-Jansen et al. 2020	Clinical	69	21.5,2.9	fMRI	N/A	Indoor	0.86
[58]	Yamashita et al. 2021	Volunteer	25	23, 1.67	NIRS	Built and Natural environment	Indoor	0.91
[59]	Song et al. 2018	Volunteer	17	21.1	NIRS	Built and Natural environment	Indoor	0.77
[60]	Lee 2017	Volunteer	18	26.7,0.7	NIRS	Built and Natural environment	Indoor	0.82
[61]	Jo et al 2019	Volunteer	29	22.3,2.1	NIRS	Built and Natural environment	Indoor	0.95
[62]	Ochiai et al. 2020	Clinical	12	36.9,11.5	NIRS	Built and Natural environment	Indoor	0.68
[63]	Yu et al. 2017	Volunteer	7	36.3,11.3	NIRS	Built and Natural environment	Indoor	0.77
[64]	Zhang et al. 2020	Volunteer	31	22.6, 1.4	NIRS	Natural environment	Indoor	0.91
[65]	Park et al. 2007	Volunteer	12	22.8,1.4	TRS	Built and Natural environment	Indoor	0.77
[66]	Joung et al. 2015	Volunteer	8	22,2.2	NIRS	Built and Natural environment	Outdoor	0.82
[67]	Song et al. 2020	Volunteer	29	21,1.4	NIRS	Built and Natural environment	Outdoor	0.86
[68]	Horiuchi et al. 2014	Volunteer	15	36	NIRS	Natural environment	Outdoor	0.86
[69]	Ulrich 1981	Volunteer	18	23	EEG	Built and Natural environment	Indoor	0.77
[70]	Elsadek et al. 2021	Elderly	34	82.9, 0.78	EEG	Built and Natural environment	Indoor	0.68
[71]	Olszewska-Guizzo et al. 2018	Volunteer	29	31,10.3	EEG	Built and Natural environment	Indoor	0.86
[72]	Jiang et al. 2019	Volunteer	50	22,1.65	EEG	Built and Natural environment	Indoor	0.73
[73]	Roe et al. 2013	Volunteer	20	30	EEG	Built and Natural environment	Indoor	0.73
[74]	Gao et al. 2019	Volunteer	116	20.7,2.13	EEG	Built and Natural environment (VR)	Indoor	0.95
[75]	Mahamane et al. 2020	Volunteer	74	23	EEG	Built and Natural environment	Indoor	0.82
[77]	Grassini et al. 2019	Volunteer	32	24.7,3.7	EEG	Built and Natural environment	Indoor	0.91
[78]	Kim et al. 2019	Volunteer	60	31	EEG	Built and Natural environment	Indoor	0.91
[79]	Elsadek et al. 2021	Elderly	34	82.9, 0.78	EEG	Built and Natural environment	Indoor	0.68
[80]	Bailey et al. 2018	Volunteer	10	20	EEG	Built and Natural environment	Hybrid	0.68
[81]	Olszewska-Guizzo et al. 2021	Volunteer	25	40,17.8	EEG	Built and Natural environment	Indoor	0.91
[82]	Chiang et al. 2017	Volunteer	180	21.4,1.81	EEG	Natural environment	Indoor	0.82
[83]	Wang et al. 2020	Volunteer	180	20.7,2.56	EEG	Natural environment	Indoor	0.68
[84]	Chang et al. 2007	Volunteer	110	Not mentioned	EEG	Natural environment (VR)	Indoor	0.82
[85]	Olszewska-Guizzo 2018	Volunteer	32	27,6.5	EEG	Natural environment	Indoor	0.86
[86]	Wang TC et al. 2020	Clinical	77	59.7	EEG	Natural environment	Indoor	0.68
[87]	Rounds et al. 2020	Volunteer	29	27.65,10.04	EEG	Built environment	Indoor	0.86
[88]	Hu et al. 2020	Volunteer	8	18.41,1.28	EEG	Built environment	Indoor	0.68
[89]	Deng et al. 2019	Volunteer	60	20.8,1.02	EEG	Natural environment	Outdoor	0.77
[92]	Herman et al. 2021	Volunteer	17	NA	EEG	Natural environment	Outdoor	0.64
[93]	Lin et al. 2019	Volunteer	240	20.2,1.76	EEG	Natural environment	Outdoor	0.95
[95]	Lin et al. 2020	Volunteer	40	20.5,1.87	EEG	Natural environment	Hybrid	0.77
[96]	Yi et al. 2021	Elderly	59	75,5	EEG	Natural environment	Outdoor	0.95
[98]	Karandinou & Turner 2018	Volunteer	10	Not mentioned	EEG	Built environment	Outdoor	0.45
[101]	Hollander et al. 2016	Volunteer	5	Not mentioned	EEG	Built environment	Outdoor	0.41
[103]	Aspinall et al. 2013	Volunteer	12	30	EEG	Built and Natural environment	Outdoor	0.59
[104]	Al-barak et al. 2017	Volunteer	10	21	EEG	Built and Natural environment	Outdoor	0.73
[105]	Hassan et al. 2018	Volunteer	60	20,1.42	EEG	Built and Natural environment	Outdoor	0.91
[106]	Chen et al. 2016	Volunteer	32	20.6,1.6	EEG	Built and Natural environment	Outdoor	0.86
[107]	Reeves et al. 2019	Volunteer	36	41,11 M	EEG	Built and Natural environment	Hybrid	0.82
[108]	Olszewska-Guizzo et al. 2020	Volunteer	22	32.9,12.7	EEG	Built and Natural environment	Outdoor	0.86
[110]	Hopman et al. 2020	Volunteer	29	25,6.76	EEG	Natural environment	Outdoor	0.91
[112]	Elsadek et al. 2019	Volunteer	25	23.5,1.5	EEG	Built and Natural environment	Outdoor	0.95
[113]	Tilley et al. 2017	Elderly	8	75.75,6.76	EEG	Built and Natural environment	Outdoor	0.5
[114]	Neale et al. 2017	Elderly	95	76,8.15	EEG	Built and Natural environment	Outdoor	0.82
[116]	Neale et al. 2020	Elderly	95	76.55,8.15	EEG	Built and Natural environment	Outdoor	0.95

In addition to the overall assessment and to facilitate the synthesis of evidence, studies were grouped according to whether they used fMRI, fNIRS, or EEG methodologies (and detailed findings were reviewed by BM and LAA, respectively).

## Results

Sixty-two studies (published between 1981 and 2021) were included in the review. More than half of the studies (*n* = 34) were published in the last three years, suggesting an emerging trend of interest in the topic.

### General characteristics of included studies

Regarding the research participants, almost all studies (*n* = 59) recruited healthy volunteers; and among these, the vast majority (95%) were adults (age ranging from 20 to 41). Only five studies focused on a more vulnerable population such as the elderly (age ranging from 75 to 83); and a minority of studies (*n* = 3) targeted clinical (adult) populations, involving patients with anxiety, gambling, or other psychiatric disorders.

To investigate how urban exposure affects the brain, published work focused primarily (95%) on the neural response evoked by certain city living features; and only a few (*n* = 3) explored a rather long- impact of urban childhood upbringing or urbanicity. In terms of experimental setting, most studies were laboratory-based (66%), but some (*n* = 18) performed their experiments in an outdoor or real-life setting. It is interesting to also note that in certain cases (*n* = 5) the experiments contemplated an “hybrid” design–i.e., the protocol included both an indoor and an outdoor session (for the urban exposure and/or for the outcome brain evaluation).

When investigating the city living exposure, almost two-thirds of the studies compared natural-either green (parks, forest, or gardens) or blue spaces (lakes or riverbanks), against built environments (including highly busy areas or quieter neighborhoods). Fifteen studies performed comparisons just between natural environments and only four focused on different built environments.

For assessing brain activity, more than half of the included studies (*n* = 37) used EEG outcome measures; moreover, fMRI (*n* = 14) or fNIRS (*n* = 11) were also commonly used. It is also important to note that the vast majority (81%) of included studies (i.e., directly measuring brain activity), further complemented their analysis with other evaluations–including self-reported psychological assessments (including, for example, stress, and perceived restorativeness scales) or other physiological signals (heart rate, blood pressure, galvanic skin response, and cortisol levels).

### Functional magnetic resonance studies

Most studies using fMRI aimed to understand the relationship between the responses in certain brain areas and the exposure to either urban built or natural environments. The presentation of such findings will be followed by the studies addressing the consequences of urban upbringing and/or city living on cognitive-emotional brain processing.

#### Functional magnetic resonance-based studies comparing urban built and natural exposure

Kim et al. (41) compared the brain activity while participants were exposed to pictures of natural landscapes (including natural parks, forests, and mountains; but not necessarily from a city environment) against urban scenes (with prominent built infrastructures, such as tall buildings). It was found that frontal (particularly in the superior and middle frontal gyrus), parietal (in the superior parietal gyrus), precuneus and anterior cingulate cortex regions were the ones more active for the natural setting (also perceived by the participants as the condition eliciting more comfort). On the other hand, urban scenery (with greater suffocating and accustomed self-ratings) elicited more activity in temporal regions (anterior temporal pole, hippocampus, and parahippocampal gyrus), occipital cortex (predominantly the middle occipital gyrus) and amygdala. A second study from the same group (42) and using similar rural and urban images found, not only similar regional activation patterns, but also observed a predominant activity in the basal ganglia–very much involved in motivational, reward and hedonic value coding, for the rural scene condition.

Rather focusing on a comparison within a particular cultural context, Seiyama et al. (43) used two kinds of landscape pictures in Japan–traditional architecture/nature (JTANs) and modern cityscapes (MCs) images. After the fMRI experiments, subjects were asked to rate the emotional valence of all trials. A negative correlation was observed between JTAN self-reporting and the right precuneus activation–suggesting that this region might be more involved in the underlying process of objective scenes evaluation than the emotional experience. Another interesting observation was the fact that viewing pleasant pictures elicited greater activation of dorsal visual pathway regions (such as the superior parietal lobule and medial occipital gyrus); whereas unpleasant landscapes predominantly activated the ventral visual pathway (including the inferior temporal and inferior frontal gyri), as well as the cerebellum. It is therefore possible that, when evaluating the pleasant landscape scenery, a higher spatial processing (e.g., location and depth)–linked to the dorsal pathway–got more prominence than the ventral visual object recognition computation (e.g., color and shape).

Neural correlates of restorative environments–either built or natural–were investigated by Martínez-Soto et al. (44), by showing to participants in the fMRI photographs considered to have a high and low restorative potential. Despite not observing significant differences in the perceived stress ratings before and after viewing both types of images, some brain regions were differently activated. Specifically, the high restorative group lead to responses in the left middle frontal gyrus, insula, and cuneus. Furthermore, in the low restorative potential group, there was a higher posterior cingulate activity, which is usually associated with endogenous attention (45). Kunh and colleagues (46) adopted a complementary approach not only by focusing on the functional connectivity between brain areas (instead of task-related regional activation) but also by matching the presented natural and built environmental images according to the dimension of perceived pleasantness (although the images used were not necessarily from an urban context). They found higher levels of connectivity when participants were watching natural scenes (compared to images with buildings), making this network activation less pronounced if the individuals’ upbringing was longer in major cities.

In line with the potential role of urban green space exposure in buffering against the development of mental illness, Bratman et al. (47) investigated whether a 90-min nature walk (versus an urban walk) decreased rumination–a maladaptive pattern of self-referential (most often negative) thought very often linked to depression. To further complement reports of the healthy participants, the authors also evaluated the fMRI activation in the sub-genual prefrontal cortex (sgPFC)–a brain region known to be particularly active during sadness, behavioral withdrawal, and negative self-reflective processes. In fact, a walk in the natural setting significantly reduced the neural activity in sgPFC (and independently of the walk-related physiological activations).

#### Functional magnetic resonance-based studies focused predominantly on exposure to natural environments

In addition to the comparative built versus natural approach, some authors focused their analysis on the qualitative properties of the natural environments. For example, Tang et al. (48) compared the restorative value of exposing healthy volunteers to four different types of landscapes images: urban, mountain, forest, and water (to note, though, that the authors did not balance the order of exposition, being the urban exposure always the first experimental condition). Results from self-report questionnaires revealed significant restorative benefits with all natural environments–with the most value being for the water and mountain scenarios, and the least for the urban images. However, fMRI analyses only showed greater activation of the cuneus when urban scenes were compared against either mountain or water landscapes. Moreover, for the latter condition there was also a significant activation of the right cingulate gyrus and the left precuneus. In another example, Zhang and colleagues (49) tried to explore whether the appreciation of images from natural landscapes differed from the artificiality of human-built gardens (controlling for aesthetic pleasantness, familiarity, and color preference). Despite some common patterns of brain response–involving the medial orbitofrontal cortex and the precuneus, the authors report activation preferences for gardens in the middle temporal gyri and middle cingulate cortex; whereas, the greater responses for natural landscapes were found in the Rolandic operculum and in the anterior cingulate cortex. Finally, the contrast between gardens and natural scenes elicited stronger activation in the inferior occipital gyrus, hippocampus, cuneus, superior parietal lobule, and supplementary motor area.

The other important question is whether brain activation matches the parametric changes in the quantity of green-space exposure (including trees, bushes, and grass). Chang et al. (50) not only aimed to clarify this, but they also tried to relate such neural activation to stress and preference-related reports. By showing participants a variety of images from various metropolitan areas (equated for luminance and chrominance) with different levels of green space density, they elicited significant activations (above and beyond lower order features) in both ventral posterior cingulate cortex (vPCC) and cuneus. Furthermore, through an effective connectivity analysis they show that vPCC has a feedforward influence on cuneus, as well as on other spatial and attentional brain regions (including the superior parietal lobule and the middle frontal gyrus). The systematic dose-dependent changes elicited in brain activity (which was paralleled by changes in stress-ratings) raise awareness for the therapeutic potential for natural environmental exposure.

Finally, there has been a more recent wave of studies that adopt a mixed-methods approach that combine epidemiological, psychological, geographic information system (GIS), and neuroimaging tools (51, 52). Such innovative work uses a hybrid approach that links real-life data collection with fMRI brain activation in laboratory-based paradigms. Tost et al. (51) aimed to unravel some to the biological underpinnings on how real-life urban green space (UGS) exposure improves human well-being. First, in a group of healthy young adults living in the city of Manheim (Germany), they found a significant within-subject positive correlation between emotional well-being (assessed with a smartphone-based electronic diary) and the GIS-quantified UGS exposure during seven consecutive days. Then, they asked participants to perform a well-known emotion-related fMRI paradigm. Interestingly, individuals with the greatest positive impact of UGS exposure on the “real-life” ambulatory emotional ratings, were the ones showing lower activation on higher-order emotion regulatory brain regions–including the dorsolateral and the dorsomedial pre-frontal cortex. In another good example, Heller et al. (52) explored the potential of geolocation tracking, experience sampling and fMRI to examine whether daily variability of physical location (i.e., the number of unique locations visited in each time) was associated to self-reported positive affect. They not only found such positive location-affect relationship, but they also observed that this effect was stronger for those individuals who exhibited a stronger functional connectivity between the ventral striatum and hippocampus–two regions, respectively related to reward (53) and novelty processing (54). These findings suggest that the environmental diversity could be a key element to provide positive affect in everyday life and ultimately a sustainability strategy for the individual well-being.

#### Functional magnetic resonance-based studies related to urban upbringing and current city-living

Urban living has been associated with an increased risk of mental health disorders and chronic physical illness, being stress a potential key mediator factor (55). In a ground-breaking study, Lederbogen and colleagues (17) used fMRI to assess whether urban upbringing and city living could impact the human brain mechanisms that regulate social stress. Participants were distributed in three groups according to whether they were living in a city (>100.000 inhabitants), a town (>10.000 inhabitants) or a rural area. Moreover, how much they spent their childhood in a city was also considered. Brain activity and its link to social stress regulation was investigated using the Montreal Imaging Stressed Task (MIST)–a well-known social stress paradigm (where participants do arithmetic tasks under time pressure and that elicits neural, cardiovascular and hormonal stress-related responses). Significant differences for the level of current city living were found in the amygdala, which increased stepwise from rural to small towns, and being the highest for city dwellers. Meanwhile, urban upbringing was rather associated with the activity in peri-genual anterior cingulate cortex (also increasing linearly with the highest activation for participants raised entirely in the city environment; and lowest for those brought up in rural areas).

Another relevant study (56) focused their work on the potential relationship between upbringing, urbanicity and the human reward-based system–more specifically to dopamine gene variations. Genetic data was collected from three independent groups and included information about catechol-O-methyltransferase, dopamine receptors D1 and D2. Both gene-related data and childhood urbanicity levels were considered as independent variables for the analysis of fMRI activation during a N-back working memory task. The results showed not only independent main effects of both variables on the middle frontal gyrus activity, but they also revealed that urban childhood upbringing interacted with each gene pattern to affect cerebral responses. Being the dopamine system and the reward circuitry so much involved in neuropsychiatric illnesses, this work suggests a genotype-phenotype pathway by which neural effects of upbringing exposure could be linked to adulthood disorders.

To further explore the links between urban upbringing, its related social stress effects and psychiatric illness, Lemmers-Jansen et al. (57) investigated the association between urbanicity and trust in both healthy and psychotic individuals. They employed a trust game paradigm, which is based on investment and repayment from another player and involving two scenarios–cooperative and unfair situations. Participants were divided in two upbringing groups, categorized as high urban (>2,500 inhabitants per km^2^) and low urban (<2,500 inhabitants per km^2^) upbringing places. They found an interaction effect linking urbanicity and cooperative condition to the amygdala activation. Higher-urban patients showed a stronger left amygdala reduction of activation than lower-urban patients during cooperative investment; and this was more pronounced in psychotic patients (than in healthy controls). Therefore, their results potentially suggest that low-urban upbringing could be a protective factor for cooperation, which then increases trust related to positive feedback, and this is reflected on amygdala activation.

### Functional near-infrared spectroscopy studies

#### Indoor functional near-infrared spectroscopy-based studies comparing urban built and natural exposure

Like the approach used in several fMRI studies described above, indoor fNIRS work also compared the neural activation to urban built and natural exposure (with images or videos). In a study by Yamashita et al. (58), healthy volunteers viewed different types of nature and built environments for 3 min while fNIRS was performed. Viewing images of nature, not only increased self-reporting of comfort and relaxation, but it also reduced the activity in the right orbitofrontal cortex–a region associated with affective processing and control. Similar pre-frontal fNIRS activation was observed in experiments using only images of specific types of natural settings, including forest landscape with metasequoia tree (versus imagery of buildings in Tokyo) (59) and visual landscapes of traditional gardens (versus images of a commercial area in Korea) (60).

Other studies considered either different stimuli or group of participants. For example, when healthy volunteers (61) and patients with gambling disorder (62) listened to forest sounds (in contrast to city noise) or watched garden video clips (compared to urban scenes) (63), their pre-frontal fNIRS response was also reduced.

Finally, a randomized controlled study examined whether greenery pictures or brief relaxation techniques (and compared to a control task where subjects stared at a fixation cross) would affect brain responses during an arithmetic task (64). Contrary to the authors’ expectations, a significant activation of frontopolar and (left) orbitofrontal cortices was found only after the brief relaxation practice; and not after viewing greenery pictures or the control task.

#### Functional near-infrared spectroscopy-based studies performed outdoor

A major advantage of fNIRS compared to fMRI is the portability, and some studies performed their measurements in a real-world scenario (65, 66). For example, Park et al. (65) tested fNIRS activation before and after a 20-min walk in a forest or a built space. The results for the forest walk corroborated the reduced activation in pre-frontal areas.

To refine the protocol of the above mentioned study involving a walk in either a forest or in the city, Joung and colleagues (66) used a similar methodology but positioned participants (for 15 min) on the rooftop of a building (to avoid some stress related to the feeling of being watched in the street by other people). Other groups also aimed to compare the fNIRS activity viewing scenery in a forest area (67); whereas others contrasted the neural responses of participants when seated in a real forest with the curtain of their tent opened, as opposed to a closed-curtain condition (68). In any case, and across the different settings, the exposure to nature induced a reduced pre-frontal fNIRS activation.

### Electroencephalography studies

The number of EEG studies conducted in the laboratory were similar to those running the data collection in an outdoor setting. Moving out of a controlled environment into a more ecological setting allows more naturalistic data collection, but at a cost of increased movement artifacts or reduced signal quality of wearable EEG devices (not to mention, the presence of other covariates sometimes difficult to control). We start by presenting the findings of laboratory-based studies, where most of the analysis focused on more conventional EEG type of analysis. Then, we will describe the EEG studies obtained in outdoor settings. Importantly, most of the latter studies used commercial devices with either a very limited number of electrodes or with manufacturer’s algorithms relating EEG evaluation directly to some emotional or brain states. The lack of knowledge about the raw EEG signals in such studies impacts on the validity of their results and on their interpretation.

#### Indoor electroencephalography-based studies comparing urban built and natural exposure

Most indoor EEG studies compared brain activity while participants viewed images of either a natural or a built environment. An old EEG spectral analysis study (with only two bipolar pairs of electrodes) was one of the first to evaluate the neurophysiology underlying the exposure to different types of landscape scenes (69). It was found that imagery of different natural environments–with water and vegetation–elicited higher mean alpha values than built environment photographs. Moreover, the alpha activity was lower for blue space scenes than for greenery vegetation ones–likely due to attention-holding properties of water views.

More recently, a study that randomly assigned participants to two different offices–one with a window opened onto a green area and another one with a window looking out onto an urban built space–similarly observed an increase in frontal and occipital alpha waves for the nature exposure scenario (70). Also exploring the view from windows but using rather photographs taken at different heights (and considering various amounts of built space and vegetation), Olszewska-Guizzo and colleagues (71) found similar greater (right-side) frontal alpha power for the highest amount of green-space–but only at a certain height of view (12th floor compared to 3rd, 6th, and 24th floors). Both studies relate the alpha activity in the brain with its association with states of alertness–higher alpha activity has been linked to lower levels of arousal and feelings of relaxation.

In another study, Jiang et al. (72) broaden not only the spectrum of EEG analysis (recording beta, delta, theta and gamma frequencies also with a 2-electrode device), but also the experimental conditions–by considering pictures of gardens, natural scenery landscape, forest, city landscape and an urban city traffic (the latter was considered the reference condition). In all frequency bands, participants presented higher values for landscape pictures than the reference condition. In a cluster analysis, Roe et al. (73) conducted a similar experiment but using a 12-channel device and a commercial software for performance metrics (that considers also spectral decompositions). It was found that exposure to “green” landscapes photographs generated greater levels of “meditation” and lower “excitement”, as opposed to urban built scenes. Interestingly, no significant differences were found among relatively similar environmental scenarios presented through VR devices–although the EEG device used for this study had a very limited electrode count (74).

Adopting an event-related EEG approach with a 14-channel device, Mahamane et al. (75) compared the brain activity on a passive oddball viewing task using scenic images of natural and built environments. Their focus was on the elicited P3 and late positive potential (LPP) responses–neural correlates of categorical differences (greater activation for rare stimuli or updating contexts) and stimulus valence/pleasantness (negative stimuli have greater positive amplitude), respectively. Despite failing to see significant effects for the P3 component, built environments elicited a significantly greater LPP activity than natural environments–supporting the views that the latter context leads to better perceived pleasantness (76). With a similar goal but using a more sophisticated methodological design and equipment, Grassini et al. (77) found that urban scenery (when compared to different types of natural scenery–such as desert, forest, snow, and water) elicited a temporally complex signal: with sustained early posterior negativity, usually associated with increased visual attentional processing; followed by an increased P3 component, potentially reflecting some higher allocation of resources or cognitive load; and finally a minor LPP-like component, differently from what was previously described. To note that for this study, urban images with faces of people or other semantic content (letters or numbers) were excluded, to avoid potential attentional bias.

Considering that the presence of people in a landscape (either built or natural) modulates the human interaction with its surroundings–particularly perceived fear at night, Kim and colleagues (78) compared the alpha and beta frequencies (with a 14-channel EEG) when subjects are exposed to images with or without people on both urban (or “grey”) or nature-dominant (or “green”) environments. More important than the higher mean alpha values elicited for the “green” conditions (like the one reported by others), a significant “Landscape type” × “Human presence” interaction effect was observed. Hence, this study highlights that the presence of people is relevant when interpreting any comparative studies about built versus natural environments.

Demographic characteristics of the participants or specific contexts could also be relevant when interpreting the potential effects on the brain of more built or natural environments. A study with an elderly population exposed to a very particular type of “green”–a forest landscape showing bamboo grove (79), confirmed the previously described increased alpha waves for natural environments observed in young adults. One must be aware when interpreting this finding that the latter bamboo landscape had some sort of semantic meaning to the cultural background of the participants (being a symbol of virtue in certain oriental communities). One study (80) related the brain activity on a 5-channel wireless EEG to cognitive testing–following a 30-min walk either in a natural outdoor environment or inside a recreational building space. The study revealed significantly increased theta waves in the frontal cortex and stronger alpha levels in occipital regions–also considered a more “meditative state”–for the outdoor sessions; and the findings of “meditation” and “relaxation” (i.e., increased alpha power across all electrodes) lasted longer after walking in nature.

Related to the recent societal challenge of COVID-pandemic, Olszewska-Guizzo et al. (81) questioned whether the exposure to natural environments during the lockdown measures could mitigate the impact on mental wellbeing and on EEG frontal alpha asymmetry (FAA)–a brain pattern that is commonly associated with positive approach/emotions. Participants also viewed videos of different types of built and natural urban public spaces before and after the pandemic. Contrary to the expected, high nature exposure during the lockdown was associated with significantly lower FAA scores (i.e., less positive emotional response), potentially because individuals who did not go out much had a more approach-related motivation for the outdoor videos. Moreover, the FAA decreased the most for the videos of residential green areas–despite containing natural elements and being technically considered a green space. The authors highlighted the importance of exploring the quality of green spaces as well as of the nature experiences in the city.

#### Indoor electroencephalography-based studies focused predominantly on exposure to natural environments

Several studies focused solely on the different features of natural landscapes and their brain effects. For example, Chiang et al. (82) investigated how participants’ exposure to photographs of nature with a variety of locations and vegetation density modulated the alpha frequency (on a 2-channel EEG headset). Greater alpha activity was observed for interior forest photos (i.e., enclosure constituting of surrounding trees and other types of vegetation), as compared to the other two settings–where patches were visually overlapped or possible to be seen from far away. No significant differences were revealed across the different vegetation densities. The authors interpreted the findings by considering that a more unified vegetation arrangement could elicit mental relaxation (as opposed to a multi-level structure). Using a similar low-density EEG device and a set of videos reproducing bamboo forests with different characteristics, one study (83) found that values for high/low beta and alpha waves decreased relatively fast after the videos started and then remained stable. Moreover, the decrease was greater for bamboo forests with a higher canopy density (i.e., the proportion of area covered by the crown of bamboo trees) and a lower tilt ratio (i.e., the ratio of the number of bamboos with an inclination angle offset from vertical greater than 45°).

Inspired by the ideas of ART (32), Chang et al. (84) explored the neurophysiological response (with a multi-modality 8-channel EEG system) using images reproducing natural restorative environments (images selected by the authors for each of the known restorativeness components: being away, extent, fascination, and compatibility). All natural-restorative environments elicited greater mean alpha values in both hemispheres, when compared against a non-viewing (or control) condition. Another study (85), also exploring similar theoretical assumptions, intended to examine the effects of 3D fixed-angle videos representing natural landscapes (rated by experts as contemplative)–i.e., green outdoor settings having long vistas, lush seemingly-wild vegetation, the presence of symbolic elements, and smooth landforms, and non-contemplative ones on brain activation patterns. They surprisingly did not find effects on alpha power (and its asymmetry) but observed greater beta power on the right temporal lobe for contemplative stimuli–which was interpreted by the authors as either a potential reflection of a more holistic perception of such natural landscapes; or a consequence of a saliency-related attention due to fascination.

In attempt to translate the above-mentioned restorative qualities of natural environments into a therapeutic context, one study randomly assigned patients with generalized anxiety disorder either to virtual natural scenes or to virtual abstract paintings (86). Their EEG alpha activity (recorded with only four channels) was assessed at baseline and after some aerobic exercise. In both exposure groups, as compared to the pre-time exercise, participants presented higher alpha levels. In addition, alpha values for the virtual natural scenery were higher–suggesting that the restorative properties of natural environments may potentially help a clinical population.

#### Indoor electroencephalography-based studies focused predominantly on exposure to built environments

Two indoor studies took advantage of virtual reality (VR) to investigate the effects of different types of built environments. Rounds et al. (87) aimed to understand which architectural features could serve as possible landmarks during urban navigation tasks and used posterior theta band activity as a signature of spatial awareness and memory. Salient buildings (i.e., those with contrasting characteristics to their surroundings) were found to elicit more of such neurophysiological signatures than non-salient buildings. Moreover, those incorporating twist designs (i.e., designs with a progressively rotated façade as it gains height) benefited from a greater theta activity effect (as well as greater self-reported visual attractiveness). The second study (88) combined immersive VR with a 5-channel EEG device to evaluate the impact of a new light rail line in the suburbs of Washington D. C.–by comparing the pre- and post- VR built projections. While self-reported evaluation about the urban features (such as the building types or height, or street characteristics) revealed a clear preference in favor of the new scenario, neurophysiological activity was harder to interpret. Rather than analyzing the raw signals, the collected brain data was categorized into six emotional states with the help of a commercial software–being this a strong reason for the findings’ limitations.

#### Electroencephalography-based studies performed outdoor

Several cases of outdoor EEG studies were performed in a real-life or more ecological contexts. Deng et al. (89), for example, explored the restorative effects of exposure to three different types of natural landscape–water, lawn, and mountain. They have also looked at various landscape elements and components of a traditional urban park. It was found that the mountain landscape showed the highest alpha mean values in the frontal region–considered by the authors as consequence of a good balance between openness and enclosure, leading to a sense of encirclement and privacy positive for relaxation (90). Such findings support other indoor research emphasizing that certain types of landscape promote health in vulnerable groups (such as elderly people) (91).

Besides some benefits associated to certain type of environmental typologies, it has been debated whether the quality of the urban green space could disparately affect individual brain responses. To address this, Herman et al. (92) compared passive recreation in informal green spaces (i.e., patches of vegetated areas scattered throughout the city which are not included in the city’s planning documents as green spaces, but provide numerous benefits to residents, including walking paths and pet areas, recreational spaces, urban agriculture lots) against traditional urban green spaces (e.g., city parks recognized or planned for recreational use by inhabitants)–in terms of well-being perception and portable EEG activity. No significant differences were observed–for frontal alpha, beta, delta, gamma, or theta oscillations–between the two exposure types. However, while comparing such green areas as a function of the different levels of human interference (e.g., paths and presence of urban furniture), the levels of alertness–reduced alpha and enhanced theta–increased for the wilder scenarios.

The human presence has also been investigated in real outdoor settings as a possible relevant factor for the behavioral dynamics of city dwellers. Lin et al. (93), conducted an experiment where participants could either walk or sit in small urban green spaces with different per capita area (i.e., with high, middle, and low population density). The results showed that in general, participants had a greater frontal beta/alpha ratio for sitting (versus walking) in such green areas–interpreted as a proxy for being more nervous and stressed (94). However, this latter effect was also modulated by the number of people surrounding the individual; being walking in a highly populated and sitting in isolation the conditions with better neurophysiological signatures. A similar experiment was performed (95), where two groups were assigned to either a walking or sitting group, which performed a high-pressure learning task and then recovered in a simulated green space. Once again, results showed that walking groups had lower beta/alpha ratio (also with more self-reports of positive valence and meditation-like experience) as compared to the sitting group. On the other hand, the latter profited from higher “focus” values, suggesting that sitting may contribute to some sort of attentional restoration. It is worth mentioning that both studies were conducted in very small areas delimited by a more unified vegetation–prompting the question of whether participants would experience even a greater effect in a richer natural landscape.

The impact of physical activity in urban green spaces has also been studied in more ecological settings. Yi et al. (96) investigated how two different “forest therapy” programs–active walking versus a resting control group–could impact elderly individuals. The results showed that active walking had an increase in alpha and beta values (from baseline). Considering that some literature suggests that alpha wave activity and beta wave power decrease in early stages of Alzheimer’s disease (97), this was interpreted by the authors as a potentially relevant finding for cognitive decline prevention.

Two studies focused on how the brain reacts to navigational tasks and specific architectural features within the urban built environment. Karandinou and colleagues (98) have recorded neurophysiological data while participants navigated between specific buildings in Portsmouth city center. Beta activity peaks were observed in critical way-finding choice points in a way that has been also seen by others (99, 100); although beta activity reduced if the places were more familiar to the subjects. The other interesting study conducted by Hollander et al. (101), tested the concept of “Cognitive Architecture” (CA), meant as “a set of principles for architecture and planning practice” (102). In their experiment they contrasted two walking sessions in the Boston city area–one in a historical ethnic neighborhood with mixed-use buildings and the other one in a reconstructed area with less architectural character. The five participants in the experiment revealed greater levels of “attentiveness” and “meditation” EEG measures while being in the historical neighborhood, than for the reconstructed one. Noteworthy, besides the use of a proprietary algorithm labeling the EEG signal into such brain states (with no access to raw data findings), results were also not statistically robust given the small sample size.

Only three outdoor studies compared the impact of free walks in the built versus natural environments. Both works by Aspinall et al. (103) and Al-Barrak et al. (104) examined, using an EEG proprietary algorithm, brain responses when subjects walked through urban green spaces versus other city areas. The first study (103) explored the urban emotional experience through three different areas in Edinburgh: a shopping street, a green space street and a busy commercial district. Participants showed less “engagement” (i.e., directed attention), “frustration”, together with an increase in “meditation”, when passing from the shopping area to the urban green space. Conversely, when moving from the green area to the commercial district the levels of “engagement” raised (although this effect size was relatively small). The second study (104) rather focused on participants moving across a cafe, a supermarket and a garden; reporting stronger “meditation” levels for the garden as compared to the supermarket. Finally, Hassan et al. (105) investigated the physiological relaxation effects of a 15-min bamboo forest walk versus a similar walk in the city. Participants showed greater frontal alpha activity when walking in the bamboo forest (and compared to the city walk), an observation that is in line with other studies where walking in green areas induced a mental state of relaxation (93, 95).

A somehow different approach was used by five other studies, which decided to seat participants in different real-world scenarios and evaluated the associated brain activity. Chen et al. (106) compared EEG recordings while in a garden versus a highway island with traffic; and more efficient and strong connectivity was found during the exposure to the natural setting. Similarly, Reeves et al. (107) compared (using an indoor space as the control condition) a natural wetland area against a traffic scene. A stronger beta activity was seen for the green area–leading the authors to suggest that an involuntary attentional shift may take part of the “fascination” process; and something also supported by Olszewska-Guizzo et al. (85). However, the authors acknowledged a substantial deterioration in the EEG signal quality (due to movement artifacts and possible electrode displacement) that could undermine the robustness of the results. In another study (108), participants were either exposed to a busy street, an urban park or a green neighborhood, and the comparison was focused on the frontal alpha asymmetry (FAA)–which is positively related to favorable emotions and negatively associated to depression (109). After adjusting for environmental conditions, higher FAA values were observed in the urban park compared to the street; an observation that suggests a possible like between nature exposure and positive mood. On the other hand, Hopman and colleagues (110) investigated the changes in averaged resting state posterior alpha power (PA)–associated with an increased external processing (111)–before, during, and after a multiday exposure to nature (which included the possibility of hiking) or to urban built environments. Participants showed lower PA while exposed to the natural condition than to the urban built. Additionally, the effects of green façades were compared as possible sources of mental relaxation by Elsadek et al. (112). Here, authors found a significant increase in alpha waves for the frontal and occipital regions when participants were exposed to a façade with climbing plants as opposed to a white wall.

Finally, three of the outdoor studies deserve particular attention as they targeted their experiments for an elderly population. Tilley et al. (113) showed, with a mixed methods approach, that participants had EEG signals compatible with a reduced attentional effort (using an algorithm and not the raw signal data) while walking in a green space versus an urban busy (and with more built environment) street. Furthermore, the level of frustration did not appear to change while transitioning from the green to the busy environment. In a relatively similar design but adding also a “quiet” urban area, Neale and colleagues (114) instead observed an increased level of “engagement” in green areas as compared to both busy commercial and quiet residential areas. In their analysis they also found evidence supporting that part of this effects could be related to a greater attentional demand from the poorest quality of paving in natural settings (this observation is particularly interesting and worth exploring given the walking vulnerability of elderly people). Alternatively, it is also possible that the drive is rather a stronger bottom-up processing linked to involuntary attentional mechanisms (115). In a repeated experiment (116) but using the actual raw EEG data, the same group observed an attentional shift neural signature- that is lower beta activity (117)–for the green setting. On the other hand, no differences in alpha activities were observed among the urban busy with green environment–despite an increase in busy urban environments as compared to the urban quiet ones. One possible explanation could be derived by the subjects’ familiarity with places, causing a more relaxed state as opposed to the less familiar quiet areas.

## Discussion

Most analyzed studies have provided a wealth of neuroscientific evidence corroborating the often-found individual self-reports–as well as prominent environmental psychology assumptions–that nature exposure and biodiversity (over heavily dense built spaces) promote positive health and well-being benefits. Our discussion aims to resume and integrate the findings, by promoting a novel neurourbanistic perspective relevant for mental or public health practitioners and policymakers involved in urban planning and design.

The urban built environment (such as buildings, city traffic scenarios, etc.) is commonly regarded as more demanding from a cognitive perspective (when compared to natural landscape). In our analysis, non-natural city-built artifacts, likely due to a more complex architectural geometry or optical dynamics, were found to engage brain areas and elicit evoked responses associated with greater cognitive (e.g., perception and attention) demand and negative emotional states. More specifically, the urban built stimuli elicited greater fMRI activation in brain areas involved in higher-order visual processing–such as the middle and inferior occipital gyri; in episodic/semantic memory, spatial navigation and object recognition–including hippocampus, parahippocampal gyrus, and anterior temporal lobe; and in both fear and stress-related responses–the amygdala. These findings provide neural substrates to the ART concept of directed attention fatigue (more linked to higher order mental functions) and, thus, support that a more complex urban built environment could provide fewer capacities to recover and restore (32).

On the other hand, exposure to nature elicited brain activation in areas more related to basic visual processing (cuneus), visuospatial perception (superior parietal gyrus), sensorial integration (insula), emotional or cognitive control (including the anterior cingulate cortex and the precuneus) and motivational behavior (basal ganglia). Such cognitive functions are more linked to simpler or “bottom-up” attentional processes of alerting and orienting, and demand relatively few mental resources. Hence, these neural correlates suggest that the interaction with natural settings, as suggested by the ART, are compatible with our intrinsic motivations and provide restorative opportunities. Interestingly, such nature-evoked activation not only seems to have a positive dose-related effect (48), but it is also modulated (with more temporal activation) by human intervention in such setting–as in the case for human-built gardens (in comparison to wild nature) (49). To note that the precuneus and the anterior cingulate cortex have also been shown to respond–as for nature–for beauty and pleasantness assessments in other visual contexts (such as paintings) (118, 119). All these observations are in favor of a greater adaptation of the visual system for recognizing natural (and ecologically or more biological elements) images (120)–in line with the “aesthetic advantage” of nature in ART (32) and the biophilia hypothesis (34). Similarly, they also contribute for a better understanding of the potential restorative properties, as well as role in emotional regulation and cognitive control of nature exposure.

The results from EEG and fNIRS corroborated the above-mentioned fMRI observations. Most EEG studies emphasized that exposure to natural settings increased conventional neurophysiological markers of quiet or relaxing wakefulness–such as the increase in alpha rhythm. Similarly, fNIRS- pre-frontal responses, often associated with mental effort or cognitive demanding states (121), were also consistently diminished in several natural settings (in contrast to the hyperactivity seen when the exposure was for urban built space). The advantages of temporal resolution and portability of EEG and fNIRS techniques allowed them to provide complementary evidence. In fact, not only different temporally-evoked EEG responses could be observed–consistent with early attentional drive elicited by urban built scenery (77); but also, the findings observed in well-controlled laboratory experiments could be translated to outdoor real-life scenarios. Interestingly, the effects across the temporal domain have not been particularly considered in most environmental psychology theories (32, 34), although it deserves further attention given the neural findings.

Another important finding that goes beyond the impact of either built or natural surroundings, was the immediate (or more direct) and later (or upbringing) neural modulation observed because of social interaction or population density. The importance of activity-setting and social context within the normative framework of ART theoretical has also been highlighted by some authors (122). Urban density has been associated to anxiety, stress, loss of perceived control and increased risk for mental health (123, 124); but the involved brain mechanisms are unknown. Overcrowding can be felt as a social stressor, inducing feelings of violation of the personal space and activation of amygdala areas linked to fear response and negative affect (125–127). Other results further described activity in other relevant emotional- and stress-related brain areas–including the amygdala, sub-genual, and medial pre-frontal cortex. This was not only seen for comparative works on built versus natural scenarios, but it was also important when looking at the city upbringing effects. Moreover, we found brain research data supporting the epidemiological evidence either arguing for an association between raising children in cities and increased risk for psychiatric illness (56) or demonstrating that green-spaces proximity during infancy promotes mental well-being (128).

Finally, some recent studies also highlighted the expected participation in the human-environmental interaction of brain structures responsible for motivation and reward-based learning–as, for example, the basal ganglia and the orbitofrontal cortex. In addition, environmental enrichment seemed to incentive explorative behavior and self-reported positive affect, which also involves reward processing brain regions (52). This work is particularly interesting as it takes advantages of computational theories on how a learning agent interacts with the environment, at the same time as it opens doors to better links with neuropsychiatric disorders (129).

### Limitations

Despite the structured search strategy in multiple databases and the specific eligibility criteria and goals, this review has limitations. First, we tried to specifically focus on the relationship between neuroscience and urban planning/design–instead of architecture and interior design, hence it is possible that some relevant work could have been excluded. Secondly, we did not perform a meta-analysis to obtain greater accuracy or improve effect sizes across the different exposures. Even if we didn’t set any initial time limit in our search criteria, the search did not contemplated studies from last year and only publications in English were considered–which may have resulted in some studies being missed. Moreover, we did not include other important city environmental factors, such as pollution, noise, or temperature. Finally, this review contemplates only studies with direct brain measurements, neglecting that stress, and emotional aspects could also be assessed indirectly by other biometric metrics.

Additionally, the reviewed studies also have limitations. While analyzing the data we have identified gender (more males) and age (preference for the young) imbalance; and few studies focused on clinical populations or had a longitudinal design (limiting causality inference). Another limitation often found was the short exposure time for the tested conditions (for example, for either the natural or built environmental scenarios), as well as the simplicity of the stimuli used–being predominantly images (which do not allow a full experience of the context). In addition, there were little attempts in the indoor studies to combine different sensorial modalities (i.e., most studies used visual stimuli, and a few used sounds), and get closer to a more realistic scenario. Regarding the tools to investigate the brain activity, several outdoor studies using EEG did not follow conventional neurophysiological analytics–but rather used proprietary (and not necessarily validated) algorithms to directly provide an emotional or behavioral outcome. Finally, the impact of potential confounding variables (e.g., noise exposure, social interactions, personality traits, and other context variables) was frequently disregarded.

### Future research

While preforming this review, one of our intentions was to raise attention and identify key priorities for future research. First, we consider important to focus more on the underlying processes bridging urban environmental exposure and mental health and well-being. The effects of nature exposure to certain psychiatric diseases have been addressed by some studies, but their physiology is still largely unknown. By contemplating more interventional and longitudinal studies, future research could advance our knowledge and will be a step forward to move from associative to causal conclusions.

Using clinical populations in more studies may also help generating stronger evidence-based recommendations for urban health decision making. Similarly, the aging challenges associated with modern societies also prompt a call for more work on the elderly population and in those within initial stages of dementia. Some architectural features attract visual attention and remain in short term memory (87), which could possibly have some benefits in subjects with spatial disorientation. In fact, the link between urban built or natural exposure, some restorative or meditative effects and neuropsychological performance has been rarely explored. A focus on vulnerable groups (not only the elderly or patients, but also migrants or other minorities) and gender differences and balance should, thus, be further explored.

While analyzing the indoor and outdoor studies, we did not find studies comparing or particularly addressing the limitations and trade-offs of both types of experimental settings. If in the future one wants to take advantage of the emerging wearable neurotechnology, such information is critical. Furthermore, no attempts have been made to obtain multimodal data–by using, for example, EEG-fMRI techniques (or EEG electrical source imaging). This could be an important validation step to allow the field to move with more confidence from lab to the street experimentation.

Despite some recent efforts highlighting the importance of individual variability (or vulnerabilities), more research is needed. We also believe that future studies should incorporate, when interpreting the neural responses, socio-economic factors (e.g., social network or educational background); as well as other individual aspects (such as personality traits or baseline levels of stress or anxiety symptoms). There is a clear need to go beyond the idea that some exposure relates to a positive or negative brain activation (and links to self-reports). And finally, only with a thorough understanding of the human-environment relationship we can better address long-term effects of certain types of childhood upbringing.

## Conclusion

The growing urbanization and climate change are major and contemporary global challenges and, among their implications in several other domains of our daily lives, they are recognized as important risk factors for mental health issues. Modern cities are complex and multifaceted entities, which involve infrastructural, social, cultural, economic, and biological aspects. Urban science is now more than ever considered as a *trans*-disciplinary field that aims to integrate novel theoretical ideas and methodological tools for better policy and decision making. Similarly, neuroscience involves many fields of research–from molecular to clinical–and the benefits of its application in real-world settings is becoming increasingly recognized. Despite being in its infancy, neurourbanism is a new field that explores the neurological or other biological underpinnings of mental states and disorders to achieve better and healthier living in urban areas. This review focused on brain research insights from the human-environment interaction to discover ways of fostering and improve the mental health and well-being of city dwellers. We collected and described in detail the wealth of evidence from fMRI, EEG, and fNIRS studies supporting how the urban environment–built or natural–can affect the neural circuits of our brain.

Our findings offer a more integrative view on how the urban built artifacts elicit greater perceptual and cognitive processing, at the same time as it expands our knowledge about the restorative potential of natural environments. The neural physiology, connectivity, and dynamics described underpins the neural substrates for theoretical constructs in environmental psychology. As a whole, our neuroscientific evidence assists urban planners, organizations, and communities to increase green and blue spaces, considering their biodiversity and quality, within the urban infrastructure–as it potentiates neural mechanisms linked to mental restoration (82) and stress-recovery (49, 108, 112). Furthermore, supporting the access to local natural landscapes (such as wetlands and forests) provide positive cognitive and emotional feedback to both healthy and more vulnerable groups (e.g., the elderly population) (89, 96, 106). Furthermore, city planning should consider overcrowding as a potential stressor (17), and take into account the space behavior in relation to per capita area when designing urban built and natural spaces (93).

Finally, more work is needed to fully embrace the underlying mechanisms linking cities and brains. The future research in the field should take advantage of the impressive modern tools to characterize behavior, neurophysiology, and environmental factors–it is an exciting time for putting neurourbanism in practice!

## Author contributions

LAA, PM, and BM defined domains. LAA, IA, and BM conducted a full-text analysis. LAA, DAB-M, and BM performed a quality assessment and analyzed the results. LAA and BM participated in writing the manuscript. LAA, IA, AB, PM, and BM contributed to assessing the relevance of the articles by abstracts and titles and analyzing and extracting data. All authors contributed to the article and approved the submitted version.
